# Occult pyogenic liver abscess in an adolescent with type 2 diabetes

**DOI:** 10.1007/s12020-013-0036-6

**Published:** 2013-08-15

**Authors:** Regina Williams, Noelle S. Larson, Jordan E. Pinsker

**Affiliations:** Division of Pediatric Endocrinology, Department of Pediatrics, Tripler Army Medical Center, 1 Jarrett White Road, Honolulu, HI 96859-5000 USA

**Keywords:** Adolescent, Diabetes, Fever of unknown origin, Kehr’s sign, *Klebsiella pneumoniae*, Liver abscess

## Abstract

Pyogenic liver abscess is a rare complication of diabetes, usually seen in adults greater than 50 years of age who have had diabetes for many years. We describe an 18-year-old male with type 2 diabetes found to have a pyogenic liver abscess caused by *Klebsiella pneumoniae*, and show accompanying images from his evaluation for fever of unknown origin (FUO). We conclude that in a child or adolescent with FUO and diabetes, occult pyogenic liver abscess must be considered.

An 18-year-old male was admitted to the pediatric intensive care unit in diabetic ketoacidosis (DKA). He arrived in the United States from the Philippines 3 days prior, where he was experiencing abdominal and shoulder pain for the last few months. On the day of admission he developed emesis and was brought to the emergency department. There he was noted to be febrile and reported night sweats, fever, malaise, and significant weight loss over the past several months.

His history was significant for type 2 diabetes mellitus (T2D) diagnosed 3 years ago while living in the Philippines. He was placed on insulin upon diagnosis, but was quickly weaned to metformin alone. He subsequently self-discontinued his metformin 2 years prior to presentation. Upon arrival to our center his hemoglobin A1c (HbA1c) was 15.8 %.

After treatment for DKA, he was transitioned to subcutaneous insulin, but continued to have fever up to 40 °C and a very large insulin requirement (1.6 U/kg/day). Extensive workup for fever of unknown origin (FUO) including blood and urine cultures, PPD, chest X-ray, viral cultures, stool cultures, malaria smears, and dengue titers were all negative. Abdominal CT scan, however, showed a heterogeneous fluid-filled mass in the right hepatic lobe (Fig. 1a). Percutaneous drainage revealed the mass to be a pyogenic liver abscess caused by *Klebsiella pneumoniae*. Following drainage of the abscess and treatment with intravenous antibiotics, the abscess cavity resolved (Fig. 1b), the patient defervesced, and his insulin resistance gradually improved. His shoulder pain ceased, suggesting this symptom was due to referred pain from diaphragmatic irritation (Kehr’s sign). He was discharged home on under 1 U/kg/day of insulin and continued to wean his dose over time.

**Fig. 1:**
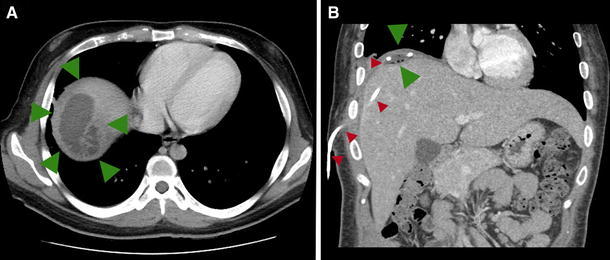
Abdominal CT-scan (**a**) showed a heterogeneous fluid filled mass in the right hepatic lobe, with thickened internal septations and partial thickening of the outer wall (*green arrows*). The mass measured approximately 7.3 × 5.3 × 4.6 cm^3^. The largest pocket of fluid measured approximately 6.1 × 3.3 cm^2^. Percutaneous drainage of the mass confirmed it to be a pyogenic liver abscess due to *Klebsiella pneumoniae*. After drainage of the abscess and treatment with intravenous antibiotics, (**b**) the abscess cavity abutting the diaphragm was almost completely cleared (*green arrows*), as shown with the drain in place (*red arrows*)

The incidence of pyogenic liver abscesses has increased over the past two decades, especially in Asia. In some studies, 50 % of reported patients with pyogenic liver abscess have diabetes [1]. Occult liver abscess has been reported as an underlying cause of FUO in patients with T2D, but this usually occurs in adults >50 years of age who have had diabetes for many years [1, 2]. *K. pneumoniae*, the underlying pathogen in this case, is a common causative organism in patients with diabetes. The hypermucoviscous K1 and K2 serotypes are largely responsible for invasive liver abscesses in this population [2]. To our knowledge, this case describes the youngest patient with diabetes to have an occult *K. pneumoniae* liver abscess causing FUO.

*K. pneumoniae* liver abscess most often presents in patients with an absence of underlying biliary disease, and typically appears as a thin-walled abscess with necrotic debris on CT scan [3]. Up to 24 % of adults have extra-hepatic complications [3]. Our patient had a thick-walled abscess and no evidence of metastatic infection, possibly related to his younger age.

Liver abscess with *K. pneumoniae* is also linked to poor glycemic control [2]. We suspect that our patient with T2D developed this abscess in relation to a prolonged period of poor glycemic control, as evidenced by his extremely elevated HbA1c and significant insulin resistance. In addition, he presented to our center in DKA, relatively uncommon for a patient with T2D.

We conclude that in a child or adolescent with FUO and diabetes, occult pyogenic liver abscess must be considered.
